# Mitochondrial genome of *Acheilognathus striatus* characterisation and phylogenetic analysis

**DOI:** 10.1080/23802359.2026.2617772

**Published:** 2026-01-24

**Authors:** Ruixin Yang, Jinhui Yu, Yongtao Tang, Chuanjiang Zhou

**Affiliations:** a College of Life Sciences, Henan Normal University, Xinxiang, Henan, People’s Republic of China; b College of Fisheries, Henan Normal University, Xinxiang, Henan, People’s Republic of China

**Keywords:** Mitochondrial genome, *Acheilognathus striatus*, Acheilognathinae, phylogenetic relationships

## Abstract

In this study, the taxonomic position of *Acheilognathus striatus* was clarified through mitogenome analysis. The circular mitogenome is 16,692 bp long and comprises 13 protein-coding genes, two ribosomal RNA genes, 22 transfer RNA genes, and one non-coding D-loop region. The mitogenome exhibits AT skewness and anti-G bias. Phylogenetic trees were constructed using 22 Acheilognathinae species, with Gobioninae and Leuciscinae used as outgroups. The phylogenetic tree revealed that *A. striatus* formed a sister group with *R. shitaiensis*. This study contributes to a better understanding of the mitogenome characteristics and evolutionary relationships among Acheilognathinae species.

## Introduction

*Acheilognathus striatus* (Yang et al. 2010) is a freshwater fish species native to the Yangtze River, characterized by long barbels and a prominent black stripe extending from the body to the caudal peduncle. The last unbranched rays of both the dorsal and anal fins are thicker than the first branched rays. *A. striatus* belongs to the subfamily Acheilognathinae of the family Cyprinidae (Yang et al. 2010). Due to its ornamental value, *A. striatus* has been heavily exploited in its natural habitats, resulting in significant population decline. However, its phylogenetic placement has not yet been clearly resolved at the molecular level.

Mitogenome data of Acheilognathinae, Gobioninae, and Leuciscinae were retrieved from the NCBI database. *Biwia yodoensis*, *Microphysogobio hsinglungshanensis*, *Gobiobotia homalopteroidea*, and *Tinca tinca* were selected as outgroups. Thirteen PCGs were concatenated, and Bayesian inference (Ronquist and Huelsenbeck 2003) and maximum likelihood analyses (Stamatakis 2006) were performed based on optimal nucleotide substitution models and best partitioning schemes (Wang et al. 2025). This study provides valuable insights into the phylogeny and evolutionary origins of Acheilognathinae species.

## Materials and methods

### Sample collection, preservation, and DNA extraction

**Figure 1.**
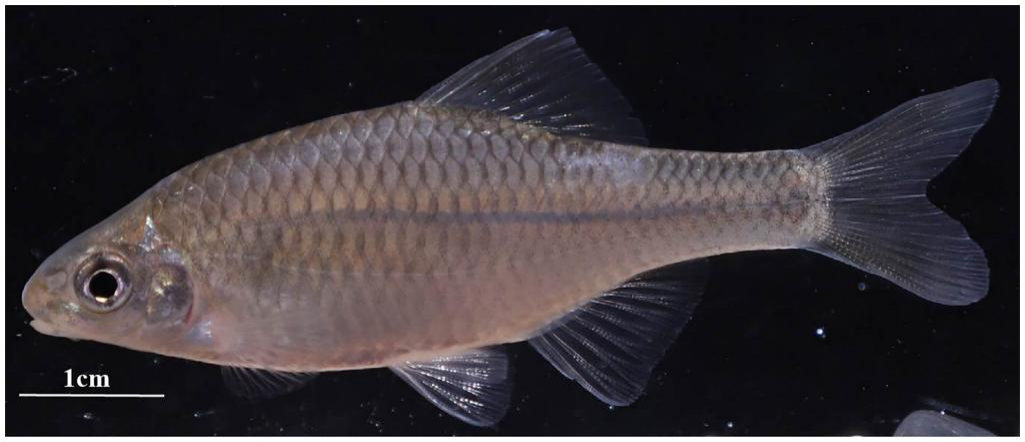
Photograph of a female *A. striatus* specimen was collected from huangshan city, Anhui province (29°43′2.1ʺN, 118°19′56.9ʺE). The image was provided by jinhui Yu.

Samples of *A. striatus* (Figure 1) were collected alive and anesthetized prior to processing. The specimens were obtained from Huangshan City, Anhui Province (29°43′2.1ʺN, 118°19′56.9ʺE). Voucher specimens were deposited at the College of Life Sciences, Henan Normal University (voucher no. 23112101; contact: Chuanjiang Zhou, chuanjiang88@163.com). Genomic DNA was extracted using the phenol-chloroform method (Gautam 2022). High-throughput sequencing at 30× coverage was performed by PersonalBio (Nanjing) Biotechnology Co., Ltd.

### Mitogenome annotation, visualization, and comparative analysis

**Figure 2.**
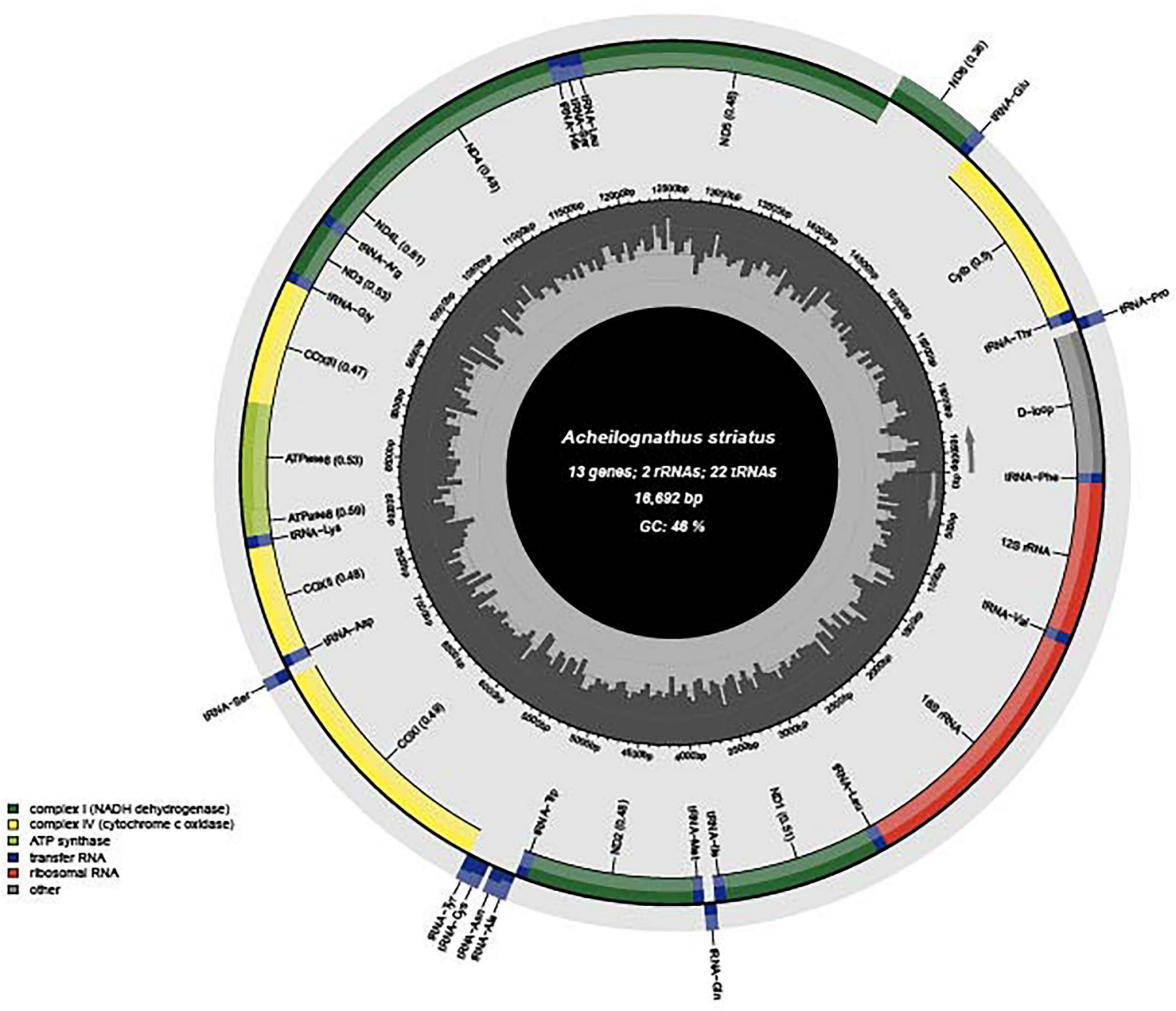
Gene map of the circular mitogenome of *A. striatus* generated with mitofish. The outer ring represents the light strand, and the inner ring represents the heavy strand. The GC content, gene composition, and length are illustrated in the center.

The mitochondrial genome of *A. striatus* was assembled and annotated from high-throughput sequencing data using GetOrganelle (Jin et al. 2020). Annotation was performed based on the codon usage of teleost fish, followed by verification of the circular conformation of the mitogenome. Mitofish was employed to analyze genomic structure, including composition, position, and gene length (Zhu et al. 2023) (Figure 2). A complete gene map of the mitogenome was constructed.

PhyloSuite v1.2.3 was used for amino acid translation, base composition analysis, and Relative Synonymous Codon Usage (RSCU) calculation (Zhang et al. 2020; Xiang et al. 2023). CodonW 1.4.2 was applied to compute the effective number of codons, codon adaptation index, codon bias index, and GC3 content (Puigbò et al. 2008) (Table S1).

### Phylogenetic analyses

To determine the phylogenetic position of *A. striatus*, 26 species from Acheilognathinae, Gobioninae, and Leuciscinae were selected from the NCBI database. *B. yodoensis*, *M. hsinglungshanensis*, *G. homalopteroidea*, and *T. tinca* were used as outgroups. Phylogenetic trees were constructed using Bayesian inference and maximum likelihood analyses in PhyloSuite v1.2.3. PartitionFinder identified the best partitioning schemes and substitution models according to the Bayesian information criterion (Penny et al. 2007). ModelFinder determined the optimal model based on the Akaike information criterion (Kalyaanamoorthy et al. 2017).

### Data resources

Complete mitogenome sequences of the 26 species were downloaded from the NCBI GenBank database (Table S2). The sequencing data for *A. striatus* were deposited in the National Center for Biotechnology Information under accession number PV469664.

## Results

### Genome structure and characteristics

The mitogenome of *Acheilognathus striatus* was found to be 16,692 bp in length, comprising 13 protein-coding genes (PCGs), 22 tRNAs, two rRNAs, and one D-loop control region, like for most other fish species. Except for eight tRNAs and the *NAD6* gene, located on the light strand, the remaining 28 fragments are encoded on the heavy strand, consistent with the typical mitochondrial gene architecture of teleost fishes (Jing et al. 2024) (Table S3). Overlaps were identified between *trnI - trnQ*, *ATPase 8* - *ATPase 6*, *NAD4L - NAD4*, *NAD5 - NAD6*, and *trnT - trnP*, with a total overlap length of 21 bp, accounting for approximately 0.13% of the genome. In addition, 13 intergenic spacers with a cumulative length of 60 bp were observed, representing 0.36% of the genome. The mitogenome displayed the highest base composition of adenine (A, 28.5%), followed by cytosine (C, 28.2%), thymine (T, 25.6%), and guanine (G, 17.7%) (Table S4). The 13 PCGs spanned 11,553 bp and encoded 3,851 amino acids. Leucine, isoleucine, threonine, and alanine were the most abundant amino acids (Figure S1). Relative Synonymous Codon Usage (RSCU) analysis (Khandia et al. 2024) revealed a strong preference for codons containing adenine and uracil bases. Several codons, particularly AUU and AAA, had RSCU values > 1, reflecting codon bias within the mitochondrial genome (Table S5).

All PCGs initiated with the start codon “ATG”, except for *COX1*, which began with “GTG”. The stop codon “TAA” was detected in *NAD1*, *COX1*, *ATPase 6*, *NAD4L*, and *NAD5*, whereas “TAG” was used by *NAD2*, *ATPase 8*, *NAD3*, and *NAD6*. In contrast, *COX2*, *COX3*, *NAD4*, and *CYTB* terminated with the incomplete stop codon “T–”.

### Phylogenetic analysis

**Figure 3.**
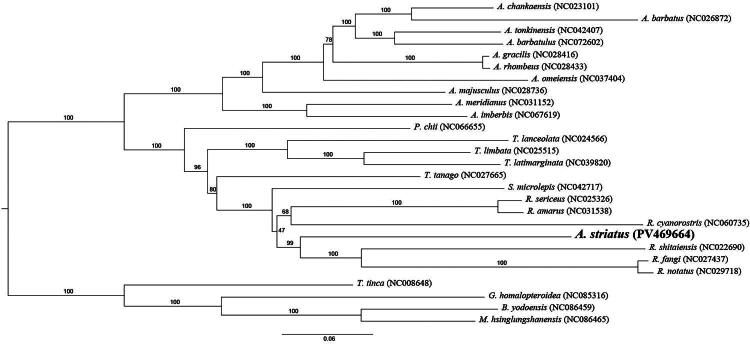
Phylogenetic trees based on ML (upper) and BI (lower) analyses. The accession numbers and citations are as follows: *A. barbatulus* (NC072602), *A. barbatus* (NC026872, Tao et al. 2016), *A. chankaensis* (NC023101, unpublished), *A. gracilis* (NC028416, unpublished), *A. imberbis* (NC067619, unpublished), *A. majusculus* (NC028736, unpublished), *A. meridianus* (NC031152, unpublished), *A. omeiensis* (NC037404, unpublished), *A. rhombeus* (NC028433, unpublished), *A. tonkinensis* (NC042407, Zhang et al. 2018), *B. yodoensis* (NC086459, unpublished), *G. homalopteroidea* (NC085316, unpublished), *M. hsinglungshanensis* (NC086465, unpublished), *P. chii* (NC066655, unpublished), *R. amarus* (NC031538, unpublished), *R. cyanorostris* (NC060735, Li et al. 2022), *R. fangi* (NC027437, unpublished), *R. notatus* (NC029718, unpublished), *R. sericeus* (NC025326, unpublished), *R. shitaiensis* (NC022690, Li et al. 2015), *S. microlepis* (NC042717, Yu et al. 2019), *T. lanceolata* (NC024566, Xu et al. 2016), *T. latimarginata* (NC039820, unpublished), *T. limbata* (NC025515, Luo et al. 2016), *T. tanago* (NC027665, Miya et al. 2015), *T. tinca* (NC008648, Saitoh et al. 2006).

**Figure 4.**
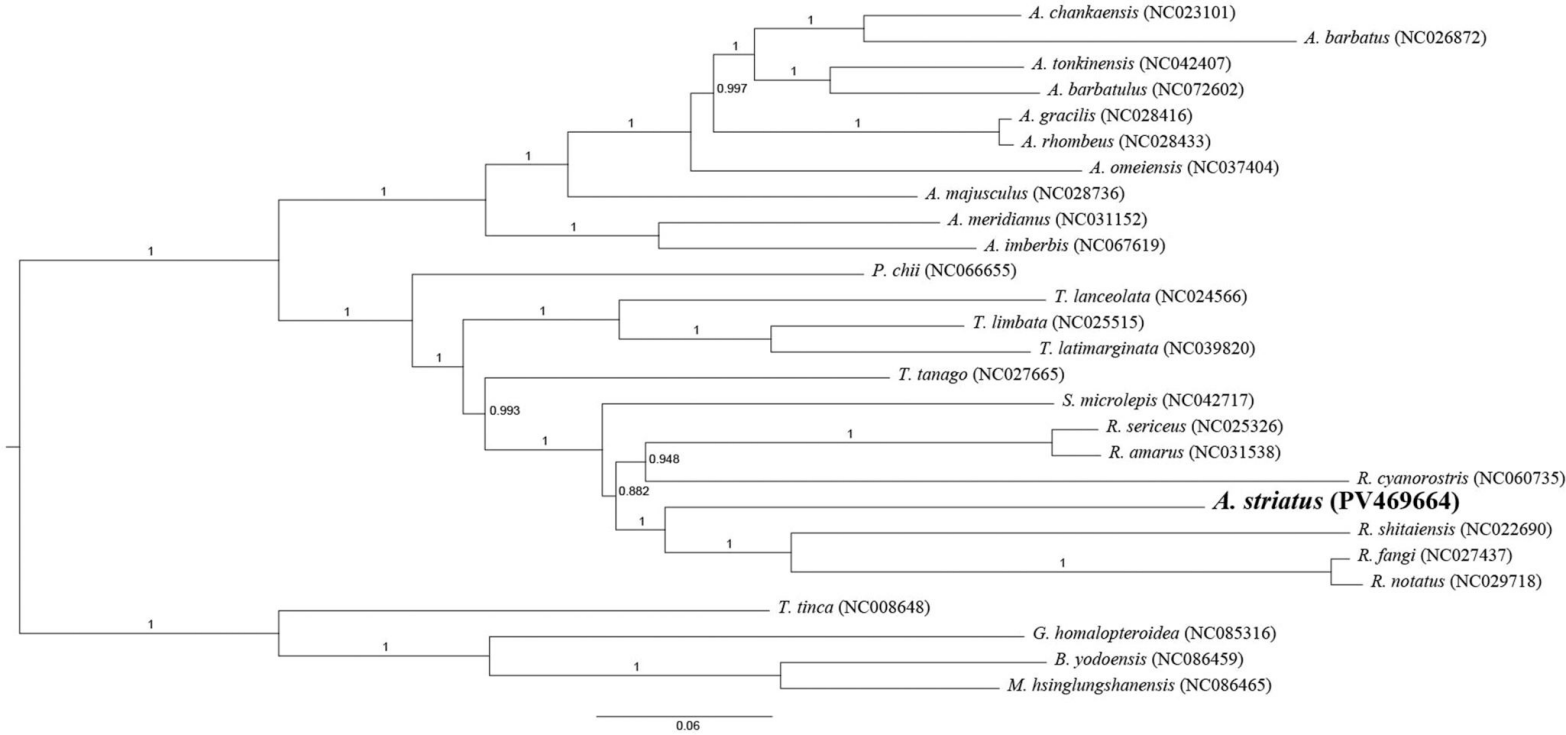
Phylogenetic tree based on 13 mitochondrial PCGs(MrBayes Tree).

Maximum likelihood (ML) and Bayesian inference (BI) trees were constructed using the concatenated sequences of 13 PCGs to determine the evolutionary placement of *A. striatus*. Both analyses produced congruent topologies. The results indicated that *A. striatus* and *R. shitaiensis* shared a closer evolutionary relationship than with other *Acheilognathus* species. The phylogenetic tree also showed that all *Rhodeus* species clustered within a single branch, and *Rhodeus* was more closely associated with *Sinorhodeus* than with *Acheilognathus*. Although few genomic studies have been performed on *A. striatus*, the data obtained here provided important baseline information for future evolutionary research (Figure 3 and 4).

## Discussion and conclusion

The complete mitogenome of *A. striatus* was successfully obtained and annotated, with a total length of 16,692 bp. Differences in mitogenome length among related Acheilognathinae species may result from variations in tandemly repeated sequences (Wang et al. 2020). The gene arrangement conforms to the typical pattern observed in Acheilognathinae (Zhu et al. 2021).

Bias and skew of base composition in mitogenome have been proposed to arise due to nucleotide mutational bias during mitogenome replication (Lakshmanan et al. 2015). The mitochondrial genome of *A. striatus* exhibited AT preference and anti-G bias, which consistent with most teleost fishes (Perna and Kocher 1995). Base composition bias was evident across several regions, with both tRNAs and PCGs showing higher AT than GC content, confirming AT preference observed in other fishes (Sun and Xu 2018). The two rRNAs displayed larger absolute AT skew and smaller absolute GC skew values (Ikemura et al. 2023), indicating strong adenine enrichment. Prior studies suggested that AT skew plays a key role in mitochondrial replication mechanisms (Sahyoun et al. 2014). AT-rich regions are also more flexible, influencing DNA bending and winding, which can affect protein binding. Analysis of base composition across the genome further revealed pronounced anti-G bias in PCGs, followed by rRNA genes, whereas tRNAs exhibited mild G bias.

The phylogenetic tree confirmed that *A. striatus* is closely related to *R. shitaiensis*, *R. fangi*, *R. notatus*, and *R. cyanorostris*. The reliability of this result was validated through comparison with previous findings (Li et al. 2022). Morphologically, Yang et al. (2010) classified *A. striatus* within the genus *Acheilognathus*, whereas molecular evidence suggests possible affinity with the genus *Rhodeus*. The lack of a clear demarcation between *Rhodeus* and *Acheilognathus* within Acheilognathinae necessitates further molecular and morphological validation.

This study presents the mitogenome sequence and structural characteristics of *A. striatus*, including its gene composition, codon usage bias, and base composition. These findings provide foundational mitochondrial genomic data for *Acheilognathus*, offering molecular insights to support species identification (Cermakova et al. 2023) and future evolutionary studies within Acheilognathinae.

## Supplementary Material

Figure S1Relative Synonymous Codon Usage (RSCU) of Acheilognathus striatus.jpg

## Data Availability

The genome sequence data that support the findings of this study are openly available in GenBank of NCBI (https://www.ncbi.nlm.nih.gov/) under the accession no. PV469664. The associated BioProject, SRA, and Bio-Sample numbers are PRJNA1277466, SRR34005014, and SAMN49108931, respectively.
