# Supplementary figures and images for: Mitochondrial genome of Acheilognathus striatus characterisation and phylogenetic analysis
Source: Mitochondrial DNA B Resour. 2026 Jan 24;11(2):285–9. doi: 10.1080/23802359.2026.2617772 (PMC12833901; doi:10.1080/23802359.2026.2617772)

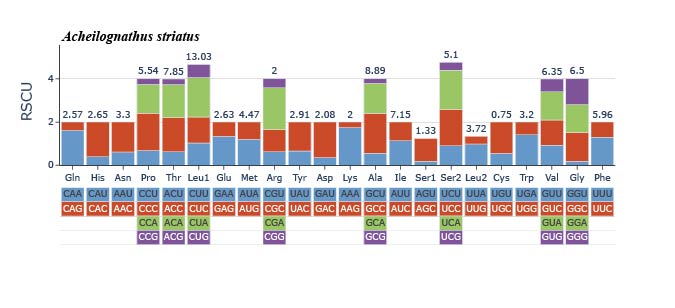

Supplement: Figure S1Relative Synonymous Codon Usage (RSCU) of Acheilognathus striatus.jpg [file TMDN_A_2617772_SM0405.jpg]
